# Supplementary material for: Groundwater as a major source of dissolved organic matter to Arctic coastal waters
Source: Nat Commun. 2020 Mar 20;11:1479. doi: 10.1038/s41467-020-15250-8 (PMC7083844; doi:10.1038/s41467-020-15250-8)
Supplement: Supplementary file 1 — Supplementary Information [file 41467_2020_15250_MOESM1_ESM.pdf]

Supplementary Information for

Groundwater as a Major Source of Dissolved Organic Matter to Arctic Coastal Waters

by Connolly et al.

**Supplementary Figures**

**Supplementary Figure 1.** Boat-towed measurements of $^{222}Rn$ concentrations around Kaktovik Lagoon conducted on August 21st and 22nd 2017. Higher $^{222}Rn$ concentrations were found adjacent to tall (~ 10 feet) eroding bluffs on the eastern side of Barter Island, where supra-permafrost groundwater (SPGW) may be greater due to steeper hydraulic gradients. Likewise, higher $^{222}Rn$ concentrations were found adjacent to a wetland along the southwestern corner of the lagoon, which may deliver relatively large amounts of SPGW during the summer.

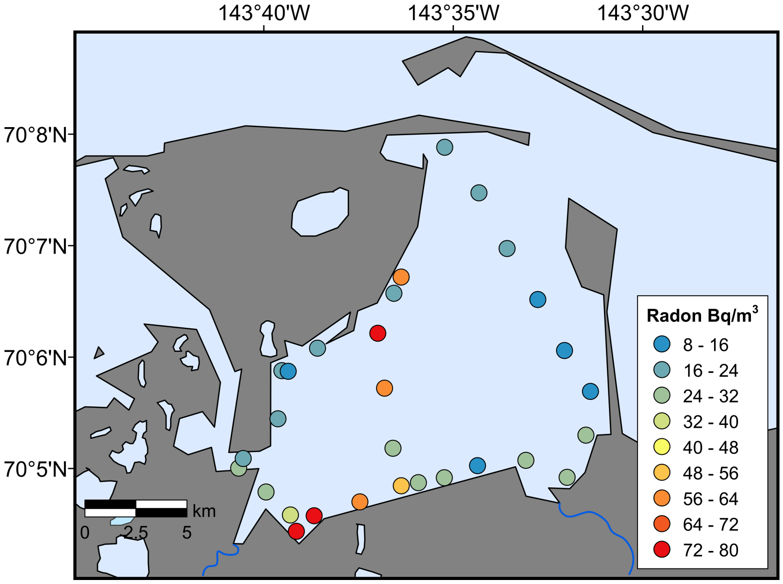


**Supplementary Note 1.**

In this section we provide a step-by-step description of the steady-state Rn isotope box model calculations that were used to estimate total groundwater discharge to Kaktovik Lagoon. This model uses the same mass balance models described in a number of other studies[1-4]. In particular, our model adopts a framework for lake systems in ref(5).

Assuming that Kaktovik Lagoon is well mixed and that lagoon Rn activity is at a steady state (i.e., $dC_{la}/dt = 0$), then only the following quantities need to be determined for the model: the mean Rn activity concentration of lagoon water $C_{la\text{-}Rn}$, a representative Rn activity concentration of groundwater inflow $C_{gw}$, the radium ($^{226}Ra$) activity concentration of the lagoon water $C_{la\text{-}Ra}$, a representative value for the Rn sediment flux $F_{sed}$, and the gas exchange flux of Rn from the lagoon surface to the atmosphere $F_{atms}$. We assume that the lagoon water level remained the same during the sampling period (21 & 21 August 2017) and so we can treat the total lagoon volume (V) and exchange with groundwater as constant. We also assume that lagoon Rn activity concentrations do not change over time. This assumption is reasonable because of the shallow and largely enclosed nature of Kaktovik Lagoon, which is expected to cause a relatively long residence time of water over the summer[6]. Given the assumptions, the Rn mass balance equation is thus:

(1) $Q_{gw} (C_{gw} - C_{la\text{-}Rn}) + F_{sed} A_{sed} - F_{atms} A_{surf} - \lambda_{Rn} V (C_{la\text{-}Rn}) + \lambda_{Ra} V (C_{la\text{-}Ra}) = 0$

$Q_{gw}$ is the unknown volumetric groundwater inflow ($m^3\ d^{-1}$), which can be calculated when all other inputs, outputs and sinks/sources are known: the input of Rn from lagoon sediments $F_{sed}$ $A_{sed}$, the loss of lagoon Rn to the atmosphere $F_{atms}$ $A_{surf}$, the loss of lagoon Rn to natural decay $\lambda_{Rn}$ V ($C_{la\text{-}Rn}$), and the introduction of Rn to lagoon water through Ra decay $\lambda_{Ra}$ V ($C_{la\text{-}Ra}$).

$C_{gw}$ (Bq $m^{-3}$) was estimated from the average of three groundwater samples (222.5 Bq $m^{-3}$). The discrete groundwater samples were analyzed using 250 ml samples following the Wat250 protocol for the RAD7 radon-in-air monitor (Durridge Company, Inc.). Each Rn measurement was corrected for $^{222}$Rn that has decayed during the time elapsed between sample collection and measurement in the field following:

(2) $C_{gw\text{-}initial} = C_{gw\text{-}final} / e^{-\lambda Rn\ t}$

$C_{gw\text{-}final}$ (Bq $m^{-3}$) is the measured groundwater Rn concentration following sample collection. $\lambda_{Rn}$ is the decay constant of $^{222}$Rn (0.181) with a half-life of 3.83 days. t (in days) is the time elapsed between each sample collection and sample Rn measurement.

$C_{la\text{-}Rn}$ (Bq $m^{-3}$) is the average of boat-towed Rn measurements collected over the course of 21 & 22 August 2017 (32.4 Bq $m^{-3}$). The measurements were done in-situ using the RAD-AQUA accessory (or water degassing chamber). Water from approximately 2-3 feet depth was pumped continuously into the RAD-AQUA accessory. The gas in the chamber was sent to three separate RAD7 units following the approach of ref(7). The RAD7 monitors each measured Rn every 30 minutes but each unit was started 10 minutes apart. With the boat moving at 1-2 knots, this allowed for traversing Kaktovik Lagoon over two days. The Rn measurements shown in Supplementary Figure 1 are located at the mid-point of a 30-minute long traverse preceding each Rn measurement.

$A_{surf}$ is the area of the lagoon surface and $A_{sed}$ is the area of the lagoon sediment surface, which are assumed to be the same. $A_{sed}$ and $A_{surf}$ ($2.47 \times 10^7\ m^2$) were estimated from the perimeter of Kaktovik Lagoon ($2.88 \times 10^4$ m) using the Google Earth polygon tool. A small embayment near a stream outlet on the eastern side of Kaktovik Lagoon was not included in this area estimate.

$\lambda_{Ra}$ is the decay constant of $^{226}$Ra ($1.19 \times 10^{-6}$) with a half-life of 1577 years.

$C_{la\text{-}Ra}$ (Bq $m^{-3}$) is the Ra concentration of Kaktovik Lagoon (5.93 Bq $m^{-3}$). We did not directly measure $^{226}$Ra in this study and so we used an estimate of late summer Ra concentration in Elson Lagoon on the western side of the Alaskan Beaufort Sea coast from ref(8) (35.6 +/− 5.8 dpm per 100 L converted to Bq $m^{-3}$). Kaktovik and Elson lagoons are functionally similar, so we assume that Ra concentrations are the same during this time period.

V of Kaktovik Lagoon ($6.15 \times 10^7\ m^3$) was estimated from $A_{surf}$ and the average lagoon depth (2.5 m), which was calculated from a NOAA bathymetric map.

The other terms for the Rn box model were calculated from the following series of equations:

(3) $F_{sed} = (D_s \lambda_{Rn})^{0.5} \times (C_{sed} - C_{la\text{-}Rn})$

$F_{sed}$ (Bq m$^2$ day$^{-1}$) is the flux of Rn from lagoon benthic sediments. $D_s$ (m$^2$ day$^{-1}$) is the wet bulk sediment diffusion coefficient. Here we assume a $D_s$ value of $4.22 \times 10^{-5}$ m$^2$ day$^{-1}$ for Kaktovik Lagoon, which is derived from the 154 cm$^2$ yr$^{-1}$ estimate for benthic sediments at 0–1 cm depth in Toolik Lake, Alaska reported by ref(9). The diffusive conditions in benthic sediments of Toolik Lake can be postulated to be similar to that in Kaktovik Lagoon because their surrounding environments are very similar. $C_{sed}$ (Bq m$^{-3}$) is the equilibrium activity of Rn measured from wet lagoon benthic sediments. $C_{sed}$ was estimated from the average of seven lagoon sediment samples collected in late August 2017 (2932 Bq m$^{-3}$). Each sediment sample was dried and measured for Rn activity in a bulk emissions chamber. Rn concentrations were then converted to the expected Rn activity from wet lagoon sediments using the equation:

(4) $C_{sed} = Mn_{Rn\text{-}box} / V_w$

$C_{sed}$ (Bq m$^{-3}$) is the expected Rn activity concentration from wet lagoon sediments in the bulk emissions chamber given a sediment porosity ($\phi_{sed}$) of 0.25 and particle density of quartz; this porosity was assumed and is typical of silty sand such as those present in Kaktovik Lagoon. $Mn_{Rn\text{-}box}$ (Bq) is the measured Rn production from a known mass of dry sediment. $V_w$ (m$^3$) is the volume of water or air in the bulk emissions chamber (2800 cm$^3$).

$F_{atms}$ (Bq m$^2$ day$^{-1}$) is the loss of Rn to the atmosphere from the lagoon water-air interface. $F_{atms}$ is calculated from the following series of equations:

(5) $F_{atms} = k(600) \times (C_{la\text{-}Rn} - \alpha\, C_{air})$

k(600) (m day$^{-1}$) is the gas transfer coefficient and $C_{air}$ (Bq m$^{-3}$) is the Rn concentration in the air, which is assumed to be 0.10. α is the Ostwald's solubility coefficient. k(600) was estimated using the following series of equations[10]:

(6) $k(600) = 0.45\, (u10^{1.6}) \times (Sc/600)^{-b}$

u10 (m sec$^{-1}$) is the wind speed at 10 m above ground and b is 0.5 for wind speeds > 3.6 m/s or = 0.667 for wind speeds < 3.6 m/s. Average wind speed above Kaktovik Lagoon (0.002 m sec$^{-1}$) was acquired from wunderground.com for 21 & 22 August 2017. Sc is the Schmidt number, which is calculated by the following equation:

(7) $Sc = v / D_m$

v is the kinematic viscosity (cm$^2$ sec$^{-1}$) and $D_m$ is the Rn molecular diffusion coefficient. v is solved by:

(8) $v = \mu / \rho$

μ (kg $m^{-1}$ $sec^{-1}$) is the absolute viscosity and ρ (kg $m^{-3}$) is the density of lagoon water with a water temperature of the 9.68 °C and salinity of 20.4 PSU. Temperature and salinity were not measured throughout Kaktovik Lagoon during the study period. Rather, these values were calculated from 15 August 2012 data reported in ref(11). Therefore we assume that these values represent ambient lagoon conditions during our sampling period. In step (7), $D_m$ is calculated by:

(9) $D_m = 10^{-(980/T)} + 1.59$

where T is the temperature of lagoon water in Kelvin (282.83 K). In step (5), α is given by:

(10) $\alpha = 0.105 + 0.405\, e^{-0.0502\, T}$

where T is the average air temperature on 21 & 22 August (6.67 °C) acquired from wunderground.com.

**Supplementary Note 2.**

Here we quantify uncertainty in total groundwater discharge by propagating standard errors (SE) for the main components of the Rn box model. These SEs reflect variability between Rn measurements as opposed to analytical uncertainty. Analytical uncertainties for Rn measurements are typically 10–15 % using these methods[4]. These components and their associated mean Rn concentrations ± 1 SE are: land-derived SPGW inputs (223 ± 20 Bq $m^{-3}$), lagoon sediment inputs (2932 ± 650 Bq $m^{-3}$), and lagoon water (32.4 ± 3.9 Bq $m^{-3}$). Propagating error from individual end-member values show (1) a 12 % increase and 10 % decrease in discharge associated with error in our groundwater Rn value; (2) a 27 % increase and decrease in discharge associated with error in our lagoon sediment Rn value; and (3) a 29 % increase and 28 % decrease in discharge associated with error in our lagoon water Rn value. Using the average Rn end-member values, our modeled groundwater discharge to Kaktovik Lagoon was $8.56 \times 10^5$ $m^3$ $day^{-1}$. The potential minimum and maximum groundwater discharge when propagating error from all three end-members is $3.50 \times 10^5$ $m^3$ $day^{-1}$ and $1.51 \times 10^6$ $m^3$ $day^{-1}$. Maximum discharge results from a propagation of error associated with the mean groundwater Rn value – 1 SE (202 Bq $m^{-3}$), the mean lagoon sediment Rn value – 1 SE (2281 Bq $m^{-3}$), and the mean lagoon water value + 1 SE (36. 2 Bq $m^{-3}$), while the opposite will result in the estimated minimum groundwater discharge.

Using the average end-member Rn values and the assumption that 5 % of total groundwater discharge measured by our Rn box model is land-derived freshwater, we estimated that SPGW exports 2128 $m^3$ of freshwater, 70.6 kg of DOC, and 4.3 kg of DON per day per km shoreline. Scaled to the Alaska Beaufort Sea coastline, SPGW delivers an estimated $4.2 \times 10^6$ $m^3$ freshwater $day^{-1}$, 138 Mg DOC $day^{-1}$, and 8.4 Mg DON $day^{-1}$. After accounting for error propagation, we estimate at a minimum SPGW exports 871 $m^3$ of freshwater, 28.9 kg of DOC, and 1.8 kg of DON per day per km shoreline. Scaled to the Alaska Beaufort Sea coastline, this equates to $1.7 \times 10^6$ $m^3$ freshwater $day^{-1}$, 56.5 Mg DOC $day^{-1}$, and 3.4 Mg DON $day^{-1}$. We estimate at a maximum SPGW exports 3753 $m^3$ of freshwater, 125 kg of DOC, and 7.5 kg of DON per day per km shoreline. Scaled up to the Alaska Beaufort Sea coastline, this equates to $7.3 \times 10^6$ $m^3$ freshwater $day^{-1}$, 244 Mg DOC $day^{-1}$, and 14.7 Mg DON $day^{-1}$.

This analysis demonstrates that we can expect an uncertainty in SPGW discharge and DOM fluxes that ranges from 76 % higher and 59 % lower than the average estimates.

**Description of Additional Supplementary Files**

File Name: Supplementary Data 1
Description: Project metadata used to generate the figures and tables in the main text and supplementary information are provided in the Supplementary Data 1 file. A description of the data columns is provided on the first tab of the Supplementary Data 1 file.

**Supplementary References**


1. Cable, J. E., Burnett, W. C., Chanton, J. P. & Weatherly, G. L. Estimating groundwater discharge into the northeastern Gulf of Mexico using radon-222. *Earth and Planetary Science Letters* **143**, 591–604 (1996).
2. Corbett, D. R., Dillon, K., Burnett, W. C. & Chanton, J. P. Estimating the groundwater contribution into Florida Bay via natural tracers, Rn-222 and CH4. *Limnology and Oceanograpy* **45**, 1546–1557 (2000).
3. Dimova, N. T. & Burnett W. C. Evaluation of groundwater discharge into small lakes based on the temporal distribution of radon-222. *Limnology and* Oceanography **56**, 486–494 (2011).
4. Dimova, N. T., Burnett, W. C., Chanton, J. P. & Corbett, J. E. Application of radon-222 to investigate groundwater discharge into small shallow lakes. *Journal of Hydrology* **486**, 112–122 (2013).
5. Kluge, T., Ilmberger, J., von Rohden, C. & Aeschbach-Hertig, W. Tracing and quantifying groundwater inflow into lakes using a simple method for radon222 analysis. *Hydrology and Earth System Science* **11**, 1621–1631 (2007).
6. Delesalle, B. & Sournia, A. Residence time of water and phytoplankton biomass in coral reef lagoons. *Continental Shelf Research* **12**, 939–949 (1992).
7. Dulaiova, H., Peterson, R., Burnett, W. C., & Smith, D.-L. A Multi-Detector Continuous Monitor for Assessment of $^{222}$Rn in the Coastal Ocean. *Journal of Radioanalytical and Nuclear Chemistry* **263**, 361–365 (2005).
8. Dimova, N. et al. Current magnitude and mechanisms of groundwater discharge in the Arctic: a case study from Alaska. *Environmental Science and Technology* **49**, 12036–12043 (2015).
9. Cornwell, J. C. & Banahan, S. A silicon budget for an Alaskan arctic lake. *Hydrobiologia* **240**, 37–44 (1992).
10. Macintyre, S., Wannikhof, R. & Chanton, J. P. Trace gas exchange across the airwater interface in freshwater and coastal marine environments. In: Matson, P.A., Harriss, R.C. (Eds.), Biogenic Trace Gases: Measuring Emissions From Soil and, Water. pp. 52–57 (1995).

11. Harris, C., McTigue, N. D., McClelland, J. W. & Dunton, K. H. Do high Arctic coastal food webs rely on a terrestrial carbon subsidy? *Food Webs* **15**, e00081 (2018).
