# Supplementary material for: Groundwater as a major source of dissolved organic matter to Arctic coastal waters
Source: Nat Commun. 2020 Mar 20;11:1479. doi: 10.1038/s41467-020-15250-8 (PMC7083844; doi:10.1038/s41467-020-15250-8)
Supplement: Supplementary file 3 — Description of Additional Supplementary Files [file 41467_2020_15250_MOESM3_ESM.docx]

**Description of Additional Supplementary Files**

**File Name: Supplementary Data 1**

**Description:** Project metadata used to generate the figures and tables in the main text and supplementary information are provided in the Supplementary Data 1 file. A description of the data columns is provided on the first tab of the Supplementary Data 1 file.
